# Multiplex Immunofluorescence Assay with Opal Reagents for Identifying Mononuclear Cell Subsets in Kidney Allograft Rejection Types

**DOI:** 10.3390/ijms262311569

**Published:** 2025-11-28

**Authors:** Ernest Kwame Adjepong-Tandoh, Jin-Myung Kim, Hye Eun Kwon, Youngmin Ko, Joo Hee Jung, Young Hoon Kim, Heounjeong Go, Haeyon Cho, Sang-Yeob Kim, Yeon-Mi Ryu, Sung Shin, Hyunwook Kwon

**Affiliations:** 1 Department of Surgery, Korle Bu Teaching Hospital, Accra P.O. Box KB-77, Ghana; 2 Division of Kidney and Pancreas Transplantation, Department of Surgery, Asan Medical Center, University of Ulsan College of Medicine, 88, Olympic-ro 43-gil, Songpa-gu, Seoul 05505, Republic of Korea; 3 Department of Pathology, Asan Medical Center, University of Ulsan College of Medicine, 88, Olympic-ro 43-gil, Songpa-gu, Seoul 05505, Republic of Korea; 4 Asan Institute for Life Sciences, Asan Medical Center, 88, Olympic-ro 43-gil, Songpa-gu, Seoul 05505, Republic of Korea

**Keywords:** kidney transplantation, antibody-mediated rejection (ABMR), T-cell-mediated rejection (TCMR), multiplex immunofluorescence (OPAL)

## Abstract

Antibody-mediated rejection (ABMR) remains a leading cause of kidney allograft failure, yet the mechanistic roles of innate immune cell subsets such as monocytes and natural killer (NK) cells remain incompletely understood. In this retrospective cohort study, we applied OPAL-based multiplex immunofluorescence (mIF) to human kidney allograft biopsies from 38 recipients with biopsy-proven ABMR (n = 19), T-cell-mediated rejection (TCMR, n = 12), or no rejection (NR, n = 7), enabling spatially resolved quantification of immune subsets in situ. Fluorescence thresholds were pathologist-validated, and co-expression phenotypes were defined using standardized segmentation and spectral unmixing. We observed a significantly higher density of CD1^4+C^D11c^+^ monocyte-derived cells in ABMR versus TCMR (*p* = 0.011), and of cytotoxic CD3^−^PAX8^−^CD16^+^CD57^+^ NK cells in ABMR versus TCMR (*p* = 0.008), implicating both subsets in ABMR pathogenesis. Spatial clustering of these populations was evident in ABMR biopsies, suggesting organized immune infiltration. A logistic regression model combining both subsets yielded an area under the ROC curve of 0.79 (95% CI: 0.65–0.93), indicating moderate discriminatory power for ABMR. While Cox regression did not reveal statistically significant associations with graft survival, CD3^−^PAX8^−^CD16^+^CD57^+^ cells showed a trend toward increased risk (HR = 2.73, *p* = 0.09). These findings support a mechanistic role for monocyte and NK cell subsets in ABMR and demonstrate the utility of OPAL mIF for high-resolution immune profiling in human allografts. Our study advances understanding of cellular immune contributors to ABMR and highlights the potential diagnostic value of intragraft mononuclear cell phenotyping.

## 1. Introduction

Kidney transplantation is the preferred treatment for end-stage renal disease (ESRD), offering improved quality of life and survival over dialysis. However, allograft rejection remains a significant challenge, with antibody-mediated rejection (ABMR) and T-cell-mediated rejection (TCMR) being the primary forms of immune-mediated graft injury. Understanding the distinct immunological profiles associated with these rejection types is essential for improving diagnostic accuracy and developing targeted therapeutic strategies [1,2].

Antibody-mediated rejection (ABMR), which is driven primarily by donor-specific antibodies (DSAs) that target endothelial cells in the graft, is a major challenge in organ transplantation. The role of T cells and B cells in rejection is well-established. T cells are key players in TCMR, whereas B cells play a crucial role in ABMR through the production of DSAs. Therefore, markers for these cells have been widely studied and are considered standard in rejection diagnostics [3,4,5].

In contrast, the roles of specific monocyte and natural killer (NK) cell subtypes in ABMR and TCMR are less well-understood. Emerging evidence suggests that these cells contribute significantly to immune responses during rejection. For example, NK cells are involved in antibody-dependent cellular cytotoxicity (ADCC) in ABMR, whereas monocytes/macrophages influence inflammatory pathways in both ABMR and TCMR [6,7].

To comprehensively study the presence and spatial distribution of these immune cell subsets within renal allografts, multiple high-dimensional immune profiling approaches have been utilized. Flow cytometry and mass cytometry (CyTOF) enable deep immune cell characterization at the single-cell level; however, these techniques lack spatial resolution as they require enzymatic dissociation of tissue into single-cell suspensions, thereby eliminating critical tissue context and cellular interactions. Single-cell RNA sequencing (scRNA-seq) has further advanced immune profiling by revealing transcriptomic heterogeneity and functional states of immune cells [8,9,10,11]. However, its reliance on gene expression data rather than direct protein detection limits its ability to fully capture post-translational modifications and immune marker co-expression patterns. Additionally, emerging high-dimensional spatial imaging platforms such as Co-Detection by Indexing (CODEX), Multiplexed Ion Beam Imaging (MIBI), and Imaging Mass Cytometry (IMC) provide an expansion of marker capacity while retaining spatial resolution but require specialized instrumentation and computational expertise for analysis [12,13,14].

To study the presence and spatial distribution of these immune cell subsets within renal allografts, we employed multiplex immunofluorescence (mIF) via Opal technology. This technique allows for the simultaneous detection of multiple immune markers on a single tissue section, providing in-depth analysis of cellular phenotypes in situ. By selecting markers for less-characterized immune cells, such as monocytes and NK cells, mIF/Opal offers an advanced method for unraveling their contributions to rejection pathophysiology. Identifying the presence, distribution, and potential role of these cell subsets in graft injury may open new avenues for targeted therapies and improve diagnostic precision in the management of kidney transplant rejection.

## 2. Results

### 2.1. Baseline Clinical and Demographic Characteristics

Table 1 shows the baseline clinical and demographic information for the 38 kidney transplant recipients who underwent for-cause kidney biopsies to evaluate different types of rejection and were included in this study. The cohort was divided into three groups based on histopathological findings: no rejection (NR, n = 7), T-cell-mediated rejection (TCMR, n = 12), and antibody-mediated rejection (ABMR, n = 19). The mean age of the participants was 43.4 ± 13.3 years, with no significant difference observed between the rejection types (*p* = 0.562). The proportion of female participants was 18.4% (n = 7), and the distribution across the groups did not differ significantly (*p* = 0.909). The average BMI was 22.5 ± 2.6 kg/m^2^, with similar values across the rejection types (*p* = 0.252). Deceased donors were involved in 28.9% of the cases, with no significant difference between the groups (*p* = 0.497). Notably, previous transplants were more common in the ABMR group (36.8%) than in the NR and TCMR groups, with a significant difference observed (*p* = 0.044), which did not hold after Bonferroni correction. The average time from transplantation to biopsy was 27.3 ± 24.4 months. This interval was significantly shorter in the NR group (9.6 ± 7.5 months) shorter than in the TCMR (21.4 ± 15.5 months) and ABMR (37.6 ± 28.3 months) groups (*p* = 0.01). The difference in time to biopsy was significant between NR and ABMR but not for other comparisons. The majority (47.4%) of the ESRD patients had an unknown etiology, followed by glomerulonephritis (GN, 15.8%), diabetes mellitus (DM, 15.8%), hypertension (HPT, 7.9%), and others (13.2%). The distribution of ESRD causes did not significantly differ between the groups. ABO-incompatible kidney transplantation was present in 23.7% of the patients, with no significant difference between the groups (*p* = 0.402). The mean serum creatinine level at the time of biopsy was 2.8 ± 1.8 mg/dL, with no significant difference between the groups (*p* = 0.98). The proportion of patients with panel-reactive antibody (PRA) levels >10% was 41.7%, and donor-specific antibodies (DSAs) were present in 50% of the patients. There was a significant difference in the presence of DSAs between the groups (*p* = 0.019), but pairwise comparisons did not reach significance after correction. Most patients were on Tacrolimus (72.2%) as a calcineurin inhibitor (CNI) at the time of biopsy, with no significant difference between the groups (*p* = 0.269). The average follow-up period after biopsy was 63.7 ± 43.1 months, with no significant difference between the groups (*p* = 0.339).

### 2.2. Histopathological Findings

**Figure 1:**
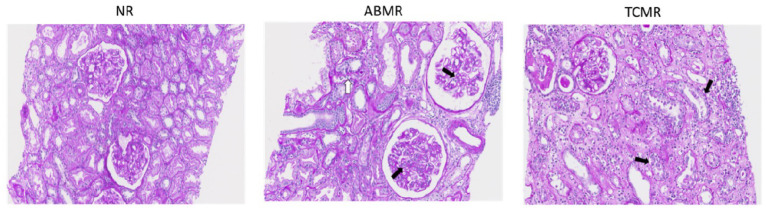
Periodic acid–Schiff (PAS) staining of representative kidney allograft biopsies classified as NR, ABMR, and TCMR at an original magnification of ×200. The ABMR biopsy demonstrates severe microvascular inflammation, with prominent mononuclear cell infiltration in peritubular capillaries (white arrow) and glomerular capillaries (black arrow), consistent with active ABMR. The TCMR biopsy exhibits high-grade tubulitis (black arrow), characterized by lymphocytic infiltration within tubular epithelium.

The histopathological characteristics of kidney transplant recipients stratified by rejection type are summarized in Table 2. The extent of C4d staining, a marker of antibody-mediated rejection (ABMR), did not significantly differ across the rejection groups (*p* = 0.610). However, interstitial fibrosis and tubular atrophy (IFTA) showed a trend towards significance, with higher severity observed in ABMR cases (*p* = 0.106). In terms of Banff scores, which assess various histopathological features, significant differences were observed in chronic interstitial inflammation (ci) and tubular atrophy (ct) scores. Peritubular capillaritis (ptc) shows a highly significant difference among the three rejection types (NR, TCMR, and ABMR). As expected, based on Banff criteria, the mean ptc score was significantly higher in ABMR (2.32 ± 0.58) compared to TCMR (1.92 ± 1.24) and NR (0.79 ± 1.07) (*p* < 0.001), reflecting the characteristic microvascular inflammation associated with antibody-mediated rejection. Figure 1 provides representative histopathological images demonstrating the characteristic differences in ci, tubulitis, and ptc among NR, TCMR, and ABMR groups.

Other Banff scores, such as v and g, did not show statistically significant differences among the rejection types. ABMR cases exhibited higher mean ci (1. 68 ± 1.11) and ct (1.63 ± 1.01) scores compared to TCMR and NR groups (*p* = 0.017 and *p* = 0.018, respectively), suggesting a more pronounced chronic injury pattern in ABMR than in TCMR and NR. A higher ptc score in ABMR signifies a greater level of microvascular inflammation, which is characteristic of this rejection type. These findings support the concept that ABMR involves a prominent humoral immune response, leading to microvascular injury in the allograft. The lack of significant differences in other Banff scores underscores the complexity and heterogeneity of rejection phenotypes in kidney transplantation.

### 2.3. Immune Cell Subsets in Kidney Allograft Biopsies

The immune cell subsets analyzed in this study were identified based on marker co-expression patterns (Table 3). The detailed absolute density values are available in Supplementary Table S1. 

**Figure 2:**
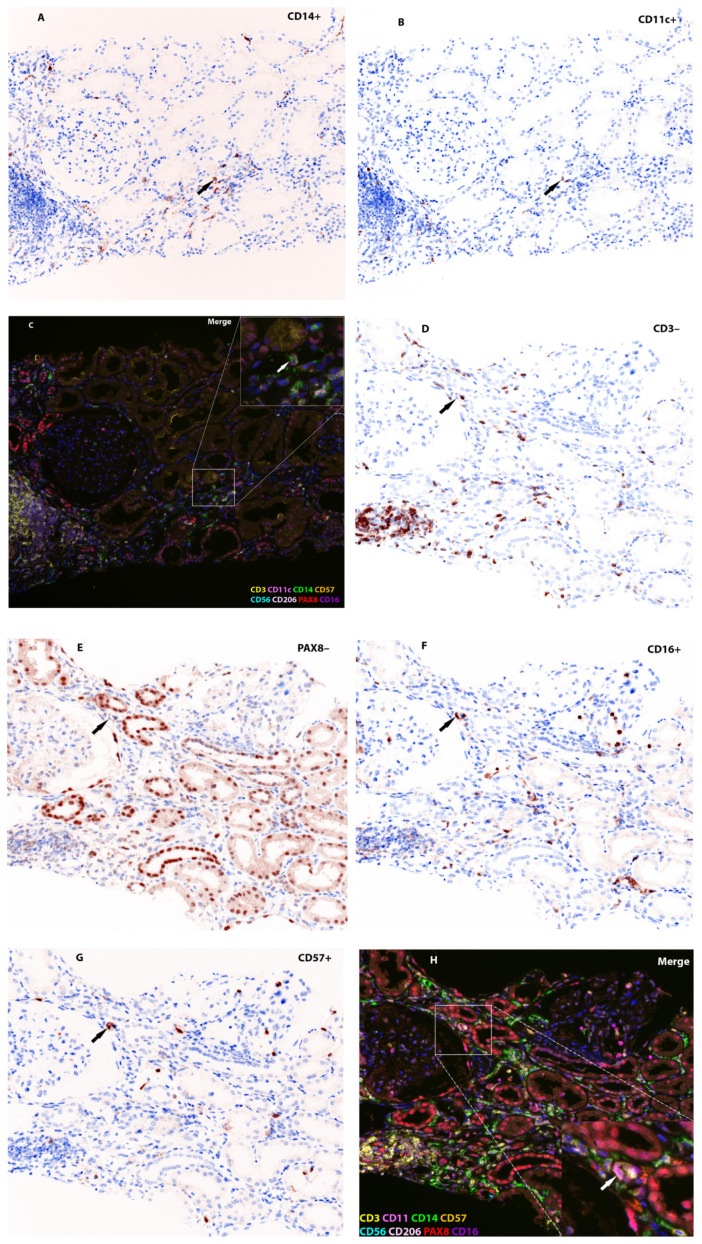
Multiplex immunohistochemistry of kidney allografts with TCMR and ABMR. Panels (**A**,**B**,**D**–**G**) show simulated brightfield images (generated by DAB transformation), while Panels (**C**,**H**) demonstrate multiplex immunofluorescence (mIF), enabling the simultaneous visualization of multiple immune markers within the same tissue section. Black arrows indicate:• CD14^+^ (**A**) and CD11c^+^ (**B**) lymphocytes in the glomerulus with TCMR.• CD3^–^ (**D**), PAX8^–^ (**E**), CD57 (**F**), and CD16^+^ (**G**) natural killer cells in ABMR. Inset: Higher-magnification composite view showing a section with CD14^+^CD11c^+^lymphocytes and CD3^–^ PAX8^–^ CD16^+^CD57^+^ natural killer cells in the glomerulus (White arrows). Original magnification: ×200. NB. DAB (3,3′-Diaminobenzidine) transformation refers to the digital conversion of fluorescence-based OPAL multiplex immunofluorescence (mIF) images into simulated brightfield immunohistochemistry (IHC)-like images.

Representative multiplex immunofluorescence images illustrating the localization of key immune markers (CD14, CD11c, CD3, CD16, CD57, and PAX8) in allograft biopsies are shown in Figure 2.

**Figure 3:**
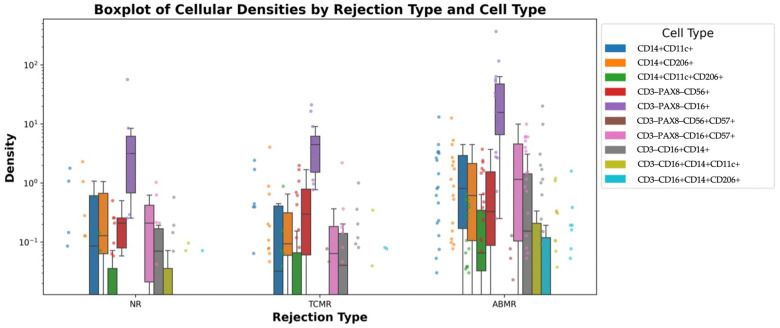
Boxplot of Mononuclear Cell Subset Densities Across Rejection Types. This plot visualizes the distribution of mononuclear cell subset densities across NR, TCMR, and ABMR groups in kidney transplant biopsies. Data points represent individual observations, with boxplots displaying the median, interquartile range (IQR), and outliers. The *y*-axis is presented on a logarithmic scale for better visualization of density variations. The Kruskal–Wallis test was used to compare cell subset densities across rejection types. Significant overall differences (*p* < 0.05) were observed for CD3^–^PAX8^–^CD16^+^CD57^+^ (*p* = 0.012), CD14^+^CD11c^+^ (*p* = 0.019) and CD3^–^PAX8^–^CD16^+^ (*p* = 0.030). Pairwise comparisons (Mann–Whitney U test with Bonferroni correction) reported significant or near-significant pairwise differences were found for: CD14^+^CD11c^+^ (NR vs. ABMR: *p* = 0.056, TCMR vs. ABMR: *p* = 0.011,• CD3-PAX8^–^CD16^+^ (TCMR vs. ABMR: *p* = 0.023),• CD3^–^PAX8^–^CD16^+^CD57^+^ (NR vs. ABMR: *p* = 0.046, TCMR vs. ABMR: *p* = 0.008 *). Asterisks (*) denote statistically significant pairwise differences at the corrected threshold (*p* < 0.0167).

The median densities of various immune cell subsets were evaluated across the three rejection types (NR, TCMR, ABMR). The results are summarized in Supplementary Table S2. The density of the CD14^+^CD11c^+^ subset significantly differed across the rejection types (*p* = 0.019). Figure 3 provides a visual representation of these differences, illustrating the interquartile ranges, medians, and individual variations in density distributions among rejection groups. The logarithmic scale enhances visibility of subtle differences between groups. This graphical representation complements the numerical data in Supplementary Table S2.

Pairwise comparisons revealed that the median cellular density of CD14^+^CD11c^+^ cells was significantly greater in the ABMR group than in the TCMR group (*p* = 0.011, after Bonferroni correction). The CD3–PAX8–CD16^+^ subset density also differed significantly across the groups (*p* = 0.03). Compared with both the NR and TCMR groups, the ABMR group had a greater density. However, the difference in the density of CD3–PAX8–CD16^+^ cells between TCMR and ABMR does did not reach the level of significance required after Bonferroni correction (*p* = 0.023). A significant difference was observed in the density of the CD3–PAX8–CD16^+^CD57^+^ subset (*p* = 0.012). Compared with the TCMR group, the ABMR group presented greater densities (*p* = 0.008 after Bonferroni correction). The densities of other immune cell subsets, such as CD14^+^CD206^+^ and CD3–PAX8–CD56^+^ cells, did not significantly differ across the rejection types after multiple comparisons were corrected. To further validate the spatial distribution of co-expressing immune subsets, we generated X/Y scatterplots using segmentation-derived centroid coordinates. As shown in Figure 2B, all segmented cells are rendered in gray, with CD14^+^CD11c^+^ cells overlaid in red and CD16^+^CD57^+^ cells in blue. In representative ABMR ROIs, these co-positive populations exhibit focal clustering within discrete tissue compartments, consistent with organized patterns of immune infiltration. In contrast, TCMR ROIs show minimal or spatially dispersed co-localization. These visual patterns complement the quantitative analyses and support the biological relevance and specificity of the marker combinations applied.

Immune cell subset densities were calculated as absolute counts per mm^2^ in each ROI. To complement this density-based approach, we also assessed subset proportions relative to total mononuclear cells, presented in Supplementary Table S3.

CD3^+^ T cell densities (Supplementary Table S4) were compared across rejection phenotypes to contextualize the discriminatory value of lineage-agnostic infiltration. A Kruskal–Wallis test demonstrated a statistically significant difference across ABMR, TCMR, and NR groups (*p* = 0.0465). Post hoc pairwise testing using Dunn’s test with Bonferroni adjustment revealed that CD3^+^ T cell density was significantly higher in ABMR compared to NR (*p* = 0.044), but not significantly different between ABMR and TCMR (*p* = 0.687) or between TCMR and NR (*p* = 0.547). These findings suggest that, although CD3^+^ quantification remains a valuable component of the diagnostic workflow, its interpretation should be integrated with clinical, serological, and additional histological parameters for accurate rejection subclassification.

We further analyzed the relative proportions of immune cell subsets, normalized to total DAPI-segmented cell counts, across rejection phenotypes. As shown in Supplementary Table S5, Kruskal–Wallis testing revealed significant differences in the proportional abundance of several subsets, including CD3^+^ T cells (*p* = 0.045), CD14^+^CD11c^+^ (*p* = 0.018), CD14^+^CD206^+^ (*p* = 0.036), and CD3^−^PAX8^−^CD16^+^ (*p* = 0.027). Post hoc Dunn’s testing demonstrated that the proportion of CD3^+^ cells was significantly higher in ABMR compared to NR (adjusted *p* = 0.038), while CD14^+^CD11c^+^ cells and CD3^−^PAX8^−^CD16^+^CD57^+^ subsets were enriched in ABMR relative to TCMR (adjusted *p* < 0.05). These findings suggest that, beyond absolute density, subset-specific representation within the tissue microenvironment may offer additional discriminatory value in characterizing rejection phenotypes.

### 2.4. Logistic Regression Analysis

Multiple logistic regression analysis was performed to evaluate the combined discriminatory power of the cell subsets CD14^+^CD11c^+^ and CD3-PAX8-CD16^+^CD57^+^ cell subsets in distinguishing ABMR from NR and TCMR. The regression model did not identify either CD14^+^CD11c^+^ or CD3-PAX8-CD16^+^CD57^+^ as statistically significant predictors of ABMR (CD14^+^CD11c^+^: OR = 1.070, 95% CI: 0.4281 to 3.219, *p* = 0.9015; CD3-PAX8-CD16^+^CD57^+^: OR = 2.732, 95% CI: 1.117 to 12.23, *p* = 0.1083). Despite the lack of statistical significance, the odds ratio for CD3-PAX8-CD16^+^CD57^+^ suggests a potential increase in the odds of ABMR with higher expression of this marker, although this finding did not reach the threshold for significance. Model fit was assessed using the Hosmer–Lemeshow test, which indicated an adequate fit (*p* = 0.9246). The classification table showed that the model correctly classified 73.68% of the cases, with a positive predictive power of 84.62% and a negative predictive power of 68.00%. Although the individual predictors were not statistically significant, the overall model suggests some potential discriminatory power in distinguishing ABMR from the combined category of NR and TCMR.

### 2.5. Receiver Operating Characteristic (ROC) Curve Analysis

**Figure 4:**
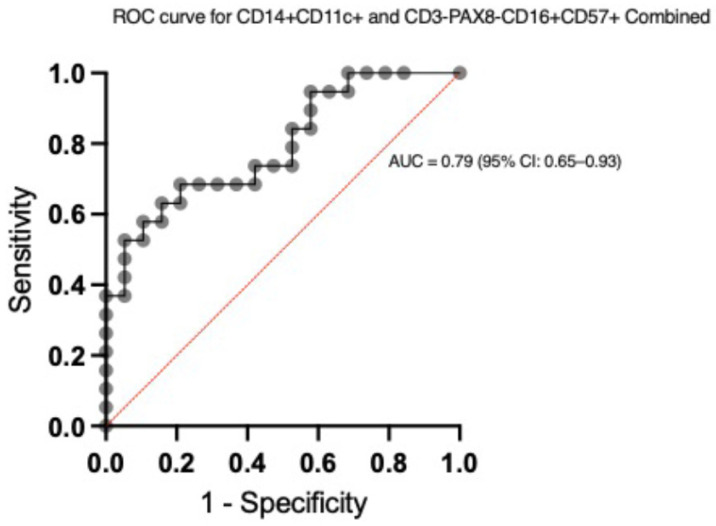
ROC Curve for the Predictive Model of ABMR vs. NR/TCMR Using CD14^+^CD11c^+^ and CD3-PAX8-CD16^+^CD57^+^ Subsets. Receiver operating characteristic (ROC) curve demonstrating the discriminatory ability of the logistic regression model in differentiating antibody-mediated rejection (ABMR) from no rejection (NR) and T-cell-mediated rejection (TCMR) in kidney transplant biopsies. The area under the curve (AUC) is 0.79 with a 95% confidence interval (CI) of 0.65–0.93, indicating moderate discrimination. The curve illustrates the balance between sensitivity and specificity across different cut-off points.

The ability of the logistic regression model to discriminate between ABMR and NR/TCMR was further evaluated using the receiver operating characteristic (ROC) curve analysis. The area under the ROC curve (AUC) was 0.7922 (95% CI: 0.6540 to 0.9341, *p* = 0.0021), indicating a fair to good level of discriminatory ability (Figure 4). An AUC of 0.79 suggests that the model is moderately effective in differentiating between ABMR and NR/TCMR cases.

### 2.6. Survival Analysis with Cox Proportional Hazards Regression

A Cox proportional hazards regression analysis was conducted to assess the impact of CD14^+^CD11c^+^ and CD3-PAX8-CD16^+^CD57^+^ cells on survival. The model included both predictors as covariates. For CD14^+^CD11c^+^ cells, the regression coefficient (β) was −0.4749 (SE = 0.5672), with a hazard ratio (HR) of 0.62 (95% CI: 0.20–1.94). The *p*-value was 0.4025, indicating that this predictor was not significantly associated with survival. For CD3-PAX8-CD16^+^CD57^+^ cells, the regression coefficient (β) was 1.003 (SE = 0.5930), with a hazard ratio (HR) of 2.73 (95% CI: 0.89–9.40). The *p*-value was 0.0908, suggesting a trend towards significance but not reaching the conventional threshold (*p* < 0.05). The model’s Akaike information criterion (AIC) was 94.08, which is slightly lower than the AIC for the null model (93.15). However, the log-likelihood ratio test comparing the full model with the null model was not significant (χ^2^ = 3.070, *p* = 0.2155), indicating that the addition of these covariates did not significantly improve the model fit. Neither CD14^+^CD11c^+^ nor CD3-PAX8-CD16^+^CD57^+^ cells were statistically significant predictors of survival. Although there was a trend indicating that higher levels of CD3-PAX8-CD16^+^CD57^+^ cells might be associated with an increased risk of the event, this was not statistically significant in this sample. The lack of significance in this model may be attributed to the small sample size (n = 38), potentially limiting the study’s power to detect a significant effect.

## 3. Discussions

In this study, we employed OPAL mIF to characterize the densities of various mononuclear cell subsets in kidney transplant biopsies.

Our findings revealed that the densities of CD14^+^CD11c^+^ and CD3-PAX8-CD16^+^CD57^+^ cell subsets were significantly greater in ABMR patients than in TCMR and NR patients. These results suggest a potential role for these cell subsets in the pathogenesis of ABMR and highlight their potential utility as biomarkers for differentiating ABMR from other forms of rejection. While earlier studies often referred to similar cells as “monocyte-derived dendritic cells” [15], more recent research suggests that CD14^+^CD11c^+^ cells in inflammatory contexts such as transplant rejection are more accurately classified as a type of monocyte-derived macrophage with some DC-like (Dendritic cell-like) properties, rather than classical dendritic cells [16]. The CD14^+^CD11c^+^ cells have been previously implicated in allograft rejection processes [17,18].

In our cohort, the density of CD14^+^CD11c^+^ cells was significantly greater in ABMR than in TCMR (*p* = 0.011), suggesting a potential role in mediating antibody-dependent cellular cytotoxicity (ADCC) in the context of ABMR. Dendritic cells are known to be potent antigen-presenting cells that can activate T cells and modulate the immune response, contributing to graft injury. The observed increase in CD14^+^CD11c^+^ cells in ABMR could indicate their involvement in presenting donor antigens and facilitating the recruitment and activation of effector immune cells, including NK cells and cytotoxic T cells, leading to graft damage [16].

Our results also revealed a significant increase in the density of CD3-PAX8-CD16^+^CD57^+^ cells in the ABMR group compared with the TCMR (*p* = 0.008). “Although NR biopsies were histologically classified as non-rejection, the modest but statistically significant difference between NR and ABMR in CD3^−^PAX8^−^CD16^+^CD57^+^ NK cells (*p* = 0.046) may reflect biological heterogeneity or low-level immune activation not captured by conventional Banff criteria. In contrast, the more robust difference between ABMR and TCMR (*p* = 0.008) supports the specificity of this NK cell subset for antibody-mediated injury.” CD3-PAX8-CD16^+^CD57^+^ cells are identified as mature NK cells with a high cytotoxic potential, that are known to mediate ADCC through their expression of CD16 (FcγRIII) [19].

The presence of these highly cytotoxic NK cells has been associated with poorer graft outcomes and increased graft dysfunction in ABMR, highlighting their potential role in graft injury [20]. The activation of these NK cells in ABMR may be driven by the engagement of their Fc receptors with DSAs bound to the allograft endothelium, leading to NK cell-mediated lysis of graft cells [21].

Although infections such as BK virus nephropathy or CMV can modulate immune cell infiltration, all included biopsies were negative for CMV and most were BK-negative. Specimens showing polyomavirus nephropathy or BK virus positivity were excluded to minimize confounding.

In the logistic regression analysis, while neither CD14^+^CD11c^+^ nor CD3-PAX8-CD16^+^CD57^+^ cells demonstrated a statistically significant independent association with ABMR, the model’s ability to differentiate ABMR from NR and TCMR was highlighted by the area under the ROC curve (AUC = 0.79; 95% CI: 0.65–0.93). These findings suggests that the combined presence of these subsets may hold discriminatory power in identifying ABMR. However, the lack of statistical significance in the Cox proportional hazards regression model suggests that while these cell subsets are associated with ABMR, their presence alone may not significantly predict graft survival. This finding emphasizes the complex nature of ABMR and the potential influence of other factors, including additional immune cells, cytokines, and genetic predispositions [22,23].

Our findings align with previous studies on the involvement of natural killer (NK) cells in antibody-mediated rejection (ABMR) but also extend these insights by incorporating key monocyte subsets. In a previous study, Jung et al. [20] demonstrated the predominance of the CD56^+^CD57^+^ NK cells subset in renal allograft biopsies with ABMR, emphasizing its important role in graft rejection. While our study identified NK cells as crucial players, particularly the CD3-PAX8-CD16^+^CD57^+^ NK subset, it further differentiated the immune landscape by assessing the involvement of other subsets, including monocytes. Specifically, CD14^+^CD11c^+^ monocytes were identified as a significant factor in differentiating ABMR from TCMR and NR.

This additional focus on monocyte subsets sets our findings apart from studies focused primarily on NK cells. Monocytes, specifically the CD14^+^CD11c^+^ subset, appear to contribute to the proinflammatory environment, working in tandem with NK cells to drive immune-mediated graft injury in ABMR. These findings suggest that both innate cytotoxic activities, as observed in NK cells, and monocyte-driven inflammation play complementary roles in the pathogenesis of ABMR.

While Legris et al. [24] highlighted the role of NK cell cytotoxicity, which is primarily mediated by DSAs, our study offers a more granular view by identifying distinct immune cell populations using OPAL mIF. This technique enabled us to delineate the complex interactions between NK cells and monocytes in ABMR, providing a broader understanding of the immune mechanisms at play.

The identification of elevated CD14^+^CD11c^+^ and CD3-PAX8-CD16^+^CD57^+^ cells in ABMR has important clinical implications. Current diagnostic criteria for ABMR rely heavily on histopathological and serological assessments, including the detection of DSAs [25,26,27,28]. Our findings suggest that profiling these cellular subsets could increase the diagnostic accuracy of ABMR, potentially enabling earlier detection and tailored therapeutic interventions to mitigate graft injury. Further studies are warranted to explore the utility of these cell subsets as part of a diagnostic panel for ABMR and to assess their potential as therapeutic targets to improve graft survival outcomes.

Density-based quantification (cells/mm^2^) was prioritized in this study to facilitate direct comparisons of immune cell infiltration across different rejection types while avoiding potential confounders introduced by variability in total mononuclear cell counts. While percentage-based analyses offer insights into immune subset composition, their dependence on total mononuclear counts can obscure true differences in infiltration. Our findings demonstrate that density-based metrics provide a more reliable assessment of immune cell distribution. However, we acknowledge the value of percentage-based analysis and have included subset proportions in Supplementary Table S6 to provide a broader immunological context.

This study has several limitations. The sample size was relatively small, which may limit the generalizability of our findings. Additionally, while our study revealed a significant association between these mononuclear cell subsets and ABMR, it does not establish a causal relationship. Future studies with larger cohorts are needed to validate these findings and to elucidate the functional roles of CD14^+^CD11c^+^ and CD3-PAX8-CD16^+^CD57^+^ cells in the pathogenesis of ABMR. Moreover, exploring the interactions between these subsets and other immune cells, such as T cells and regulatory macrophages, could provide deeper insights into the mechanisms driving ABMR and identify novel therapeutic targets.

Furthermore, although we applied validated segmentation algorithms and intensity thresholds to define immune cell subsets, we acknowledge that we did not generate spatial overlay images of cells just above threshold values. These overlays were not created as part of the initial image analysis workflow, which focused on phenotypic quantification rather than spatial visualization of individual cell coordinates. Even though we have provided scatterplots showing spatial localization of co-positive immune subsets in representative ROI in this work, the integration of spatial co-expression overlays will be an important goal in future work, particularly to enhance the interpretability and visual confirmation of immune subset localization in situ.

We also acknowledge that calculating immune subset frequencies as a proportion of total nucleated cells, rather than mononuclear immune cells alone, would offer a more holistic representation of tissue composition. While our analysis focused on high-resolution phenotyping within the immune compartment, future studies should consider integrating nuclear segmentation and total cell quantification to enhance interpretability and account for acellular areas or stromal dominance in biopsy specimens.

In this study, we carefully selected markers to focus on immune cell subsets that play a key role in rejection, particularly mononuclear cell populations. While we did include CD3^+^ T cells, our primary goal was to use co-expressing markers to identify more specialized immune subsets, rather than broadly counting T cells. Similarly, we incorporated PAX8 to distinguish epithelial structures, but we did not analyze it alongside CD3 in this dataset. Moving forward, future studies could expand the marker panel to include additional immune and epithelial markers, which would provide a more detailed understanding of immune-epithelial interactions in rejection.

Due to the retrospective nature of the study and limitations in historical pharmacy records, detailed immunosuppressive dosing data, including median cumulative doses and drug trough levels, were not consistently available across the cohort. This precluded correlative analysis between drug exposure and immune cell phenotype, and must be acknowledged as a limitation.

Furthermore, while the histological classification relied on Banff 2017, the lesion scoring components were aligned with the Banff 2013 and 2015 criteria. Given the ongoing evolution of Banff classification [29], including emerging thresholds for chronic and borderline lesions, our findings should be cautiously interpreted in the context of future schema refinements.

It should also be noted that the high proportion of patients with ESRD of unknown etiology may represent a source of immunological heterogeneity. It is possible that undiagnosed immune-mediated kidney diseases contributed to baseline immune cell alterations, which could confound rejection-specific phenotypes.

## 4. Methods and Materials

We conducted a retrospective cohort study of kidney transplant recipients who underwent diagnostic allograft biopsies for clinical indications at Asan Medical Center, Seoul, from May 2015 to December 2016. Eligible patients were adult kidney transplant recipients who presented with clinical indications for biopsy, such as proteinuria or deterioration in renal function. Pediatric kidney transplant recipients and multiple solid organ transplants recipients were excluded from the study.

### 4.1. Ethical Considerations

Written informed consent was obtained from all participating recipients. This study was conducted in accordance with the Declaration of Helsinki and was approved by the Institutional Review Board of Asan Medical Center (approval number: 2015-0758; approval date: 18 June 2015).

Due to the study’s retrospective design, the IRB waived the need for informed consent from kidney donors. We recorded and analyzed the baseline characteristics and clinical data of the recipients. All study procedures were conducted in accordance with relevant ethical guidelines and regulations. No organs or tissues were retrieved from prisoners. Living donor nephrectomies were exclusively performed at Asan Medical Center. Deceased donor kidneys (11 in all) were sourced from several hospitals across South Korea. The procurement and allocation of deceased donor kidneys were managed by the Korean Network for Organ Sharing (KONOS), ensuring compliance with ethical standards and the prohibition of organ trade or illegal distribution.

### 4.2. Immunosuppression

Induction immunosuppressive protocols differed among the participants. Basiliximab, an IL-2 receptor antagonist, was administered to 35 patients. Rabbit anti-thymocyte globulin (Thymoglobulin^®^, Genzyme, Cambridge, MA, USA) was used in 2 patients, and Daclizumab was used in 1 patient. All participants received maintenance immunosuppressive therapy consisting of calcineurin inhibitors, mycophenolic acid, and prednisolone.

### 4.3. Histopathology

Biopsy adequacy was ensured by including at least one artery and seven glomeruli per sample for rejection diagnosis and classification. The samples were subjected to thorough staining, utilizing several techniques, including Periodic Acid–Schiff (PAS), Hematoxylin and Eosin (H&E), Masson Trichrome, and Jones Methenamine Silver.

C4d immunohistochemistry (1:100, rabbit polyclonal; Cell Marque, Rocklin, CA, USA) was conducted on formalin-fixed, paraffin-embedded specimens using Ventana Medical Systems (Tucson, AZ, USA) per manufacturer guidelines [30].

All biopsy samples were assessed by two experienced renal transplant pathologists (YM Cho and H Go), blinded to clinical outcomes, using the Banff 2017 criteria as the overarching framework [28,31]. Within this, they referenced Banff 2015 definitions for the classification of rejection types, Banff 2013 scoring metrics for semi-quantitative lesion grading, and Banff 2017 definitions for interstitial fibrosis and tubular atrophy (IFTA) and inflammation in atrophic areas (i-IFTA).

Biopsies without pathological features of kidney damage were classified as normal. Discrepancies (≥1-point difference in any Banff component) were resolved by consensus review using a multi-headed microscope. If unresolved, a senior third pathologist was consulted. Final scores used in statistical analyses reflected consensus determinations. Detailed individual Banff scores are provided in Supplementary Table S7.

All biopsies underwent immunohistochemical staining for BK virus, and cases with positive staining or histopathologic features consistent with polyomavirus nephropathy were excluded. No patients had documented CMV viremia or histologic evidence of pyelonephritis.

### 4.4. Opal Multiplex Immunohistochemistry

#### 4.4.1. Sample Preparation

The OpalTM multiplex kit (PerkinElmer®, Akoya Biosciences, Marlborough, MA, USA) was employed for a fully automated seven-color immunohistochemistry method on the Leica Bond Rx™ Automated Stainer (Leica Biosystems, Nussloch GmbH, Nussloch, Germany). The automated stainer was used at the Optical Imaging Core Laboratory of the Asan Institute for Life Sciences, Asan Medical Center. Formalin-fixed paraffin-embedded (FFPE) tissue blocks from selected H&E biopsy slides (prepared from four-micron thick slices of tissue) were used. The sample slides were heated at 60 °C for forty minutes in a dry oven before being transferred to a Leica Bond Rx™ automated stainer (Leica Biosystems, Buffalo Grove, IL, USA) [20].

#### 4.4.2. Deparaffinization and Rehydration

Deparaffinization was carried out with Leica Bond Dewax solution (Cat #AR9222, Leica Biosystems).

#### 4.4.3. Antigen Retrieval

Antigen retrieval was performed with Bond Epitope Retrieval 2 (Cat #AR9640, Leica Biosystems) for 30 min. After antigen retrieval, the slides were incubated with primary antibodies followed by secondary horseradish peroxidase-conjugated polymer. Each horseradish peroxidase-conjugated polymer created a covalent bonding of a different fluorophore using tyramide signal amplification (TSA). This covalent bond reaction was followed by additional antigen retrieval with Bond Epitope Retrieval 1 (Cat #AR9961, Leica Biosystems, Milton Keynes, UK) for 20 min to remove prior primary and secondary antibodies before the next step in the sequence. Each slide was subjected to eight sequential rounds of staining. After sequential reactions, the sections were counterstained with Spectral DAPI and mounting with HIGHDEF^®^ IHC fluoromount (Enzo Life Sciences, Farmingdale, NY, USA). Details of the reagent volumes, incubation times, and conditions for multiplex immunofluorescence are provided in Supplementary Table S8. A comprehensive list of reagents, buffers, and their sources used for multiplex immunofluorescence is presented in Supplementary Table S9. The primary antibodies were freshly diluted in antibody diluent before each run and stored at 4 °C. The TSA fluorophores were prepared immediately before use according to the manufacturer’s protocol. Blocking and washing buffers were stored at room temperature and used within their validated shelf-life. Antigen retrieval buffers were preheated before use and discarded after each staining batch.

#### 4.4.4. Staining Protocol

The sections were stained using the Opal Polaris 9-Color Automated IHC Detection Kit (Akoya Biosciences, Marlborough, MA, USA). Cells were stained with antibodies against CD3 (1:5, Ventana, Tucson, AZ, USA), CD11c (1:500, Abcam, Cambridge, UK), CD14 (1:100, Abcam), CD57 (1:100, Invitrogen, Carlsbad, CA, USA), CD56 (1:100, Leica Biosystems, Newcastle Upon Tyne, UK), CD206 (1:100, R&D Systems, Minneapolis, MN, USA), PAX8 (1:100, Cell Marque, clone MRQ-50, Rocklin, CA, USA), and CD16 (1:100, Cell Marque, clone 116R), as depicted in Table 4.

The antibodies used for multiplex immunofluorescence staining, including their clones, sources, and dilutions, are detailed in Supplementary Table S10.

The fluorescence signals were captured using the following Opal fluorophores: Opal 480, Opal 520, Opal 540, Opal 570, Opal 620, Opal 650, Opal 690, and Opal 780. The sequential staining order, fluorophore assignments, and dilution factors are provided in Table 5.

After sequential reactions, sections were counterstained with Spectral DAPI (AKOYA Biosciences) for nuclear visualization and mounted using HIGHDEF^®^ IHC fluoromount (Enzo Life Sciences, Farmingdale, NY, USA). This mounting medium was selected for its anti-photobleaching properties, ensuring long-term fluorescence stability. Slides were allowed to cure before imaging [20].

### 4.5. Image Acquisition and Quantitative Image Analysis

#### 4.5.1. Multiplex Staining and Scanning

The Vectra^®^ Polaris Automated Quantitative Pathology Imaging System (PerkinElmer^®^) was used to scan multiplex-stained FFPE tissue slides and capture multispectral images for all target markers [20].

#### 4.5.2. Multispectral Library and Image Unmixing

Multiplex stained slides were scanned on Vectra^®^ Polaris Automated Quantitative Pathology Imaging System (Akoya Biosciences, Marlborough, MA, USA; Menlo Park, CA, USA) and images were visualized in Phenochart whole slide viewer (Akoya Biosciences, Marlborough, MA, USA; Menlo Park, CA, USA). Representative staining results are shown in Figure 2. The spectral unmixing process was performed using InForm^®^ 2.4 software (Akoya Biosciences, Marlborough, MA, USA), where a spectral library was constructed using single-antibody control slides to define the individual spectral profiles of each Opal fluorophore. This allowed for accurate unmixing while compensating for tissue autofluorescence. The unmixing parameters, including spectral peak assignments and autofluorescence removal, were validated prior to analysis.

#### 4.5.3. Image Visualization and Analysis

Multiplex-stained slides were analyzed using InForm^®^ 2.4 image analysis software (Akoya Biosciences, Marlborough, MA, USA) for cell segmentation and phenotype identification, and Spotfire^®^ software (https://www.spotfire.com/, 23 November 2025) (TIBCO Software Inc., Palo Alto, CA, USA) for downstream quantitative analysis. Composite fluorescence images were generated using InForm’s proprietary algorithm, and unmixed data were exported for quantification.

Cell segmentation was performed based on DAPI-stained nuclei, which served as positional anchors for identifying individual cells. Cells were classified as positive for specific markers if their fluorescence intensity exceeded pre-defined thresholds and the signal localized to the expected cellular compartment (e.g., membrane for immune markers). These fluorescence thresholds were first established using single-plex controls and histogram analyses to separate true signal from background autofluorescence. Final thresholds were pathologist-validated to ensure biological relevance. Spectral unmixing of Opal fluorophores was conducted using a custom Vectra^®^ Polaris spectral library, ensuring accurate signal discrimination.

To maintain analytical consistency, thresholds for marker positivity and segmentation parameters were standardized across all biopsies. All regions of interest (ROIs) selected for each case were pooled, and cell densities were calculated as total positive cell counts divided by the total ROI area (cells/mm^2^), making the unit of replication the entire biopsy rather than individual ROIs.

**Figure 5:**
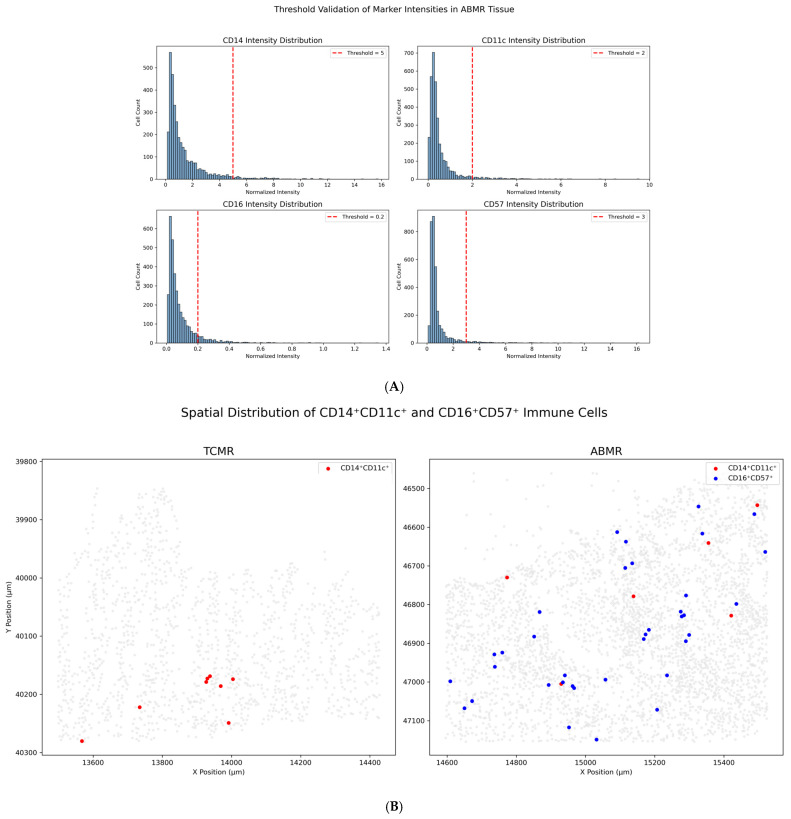
(**A**): Threshold validation of marker intensity distribution. Histograms showing the distribution of normalized fluorescence intensities for CD14, CD11c, CD16, and CD57 across all segmented cells in representative ROIs. Red dashed lines indicate the marker positivity thresholds used throughout the study (CD14 > 5, CD11c > 2, CD16 > 0.2, CD57 > 3). These thresholds were defined in collaboration with the core laboratory and validated using control tissue. (**B**): Spatial localization of co-positive immune subsets in ABMR and TCMR. Scatterplots showing the spatial distribution of segmented cells in representative regions of interest (ROIs) from TCMR and ABMR biopsies. Gray points indicate all segmented cells. CD14^+^CD11c^+^ cells are shown in red, and CD16^+^CD57^+^ cells in blue. Co-positive immune subsets are spatially clustered in ABMR tissue but sparse in TCMR, supporting threshold specificity and phenotypic validity.

To validate the robustness of the thresholding approach, we performed two complementary analyses. First, histograms of normalized fluorescence intensity were generated for each marker (e.g., CD14, CD11c, CD16, CD57), with vertical dashed lines denoting threshold cutoffs (Figure 5A). These plots demonstrated clear positive tails above background, confirming the specificity of the thresholds. Second, spatial scatterplots of segmented cells were constructed using centroid coordinates, with color overlays highlighting CD14^+^CD11c^+^ and CD16^+^CD57^+^ double-positive cells (Figure 5B). In ABMR biopsies, these populations formed focal clusters, whereas TCMR samples exhibited dispersed or absent co-localization—further supporting the biological validity of our phenotypic definitions.

**Figure 6:**
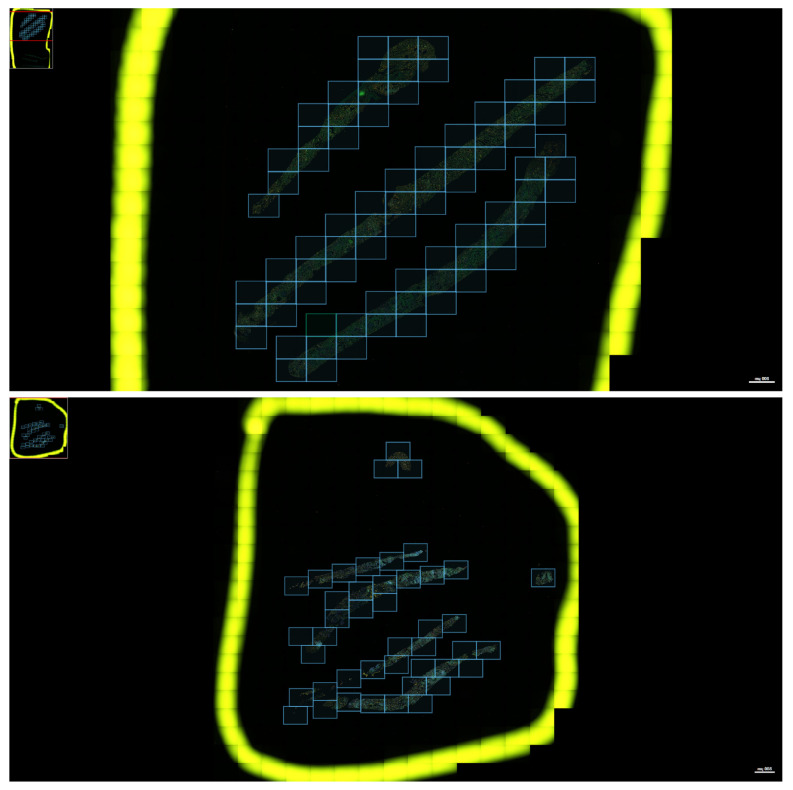
Selection of regions of interest (ROIs) for multiplex immunofluorescence analysis. Representative images of selected ROIs from kidney transplant biopsy samples. Blue boxes indicate individual ROIs selected for quantification within the tissue sections, outlined in yellow.

Regions of Interest (ROIs) were delineated using Phenochart™ (version 1.1.0) software (Akoya Biosciences, Marlborough, MA, USA), part of the PhenoImager™ HT system (formerly Vectra® Polaris—Akoya Biosciences, Marlborough, MA, USA). ROIs were selected to comprehensively cover viable cortical regions, while excluding areas with necrosis, fibrosis, or artifacts (Figure 6). The number of ROIs varied based on biopsy size and tissue quality, ensuring adequate sampling of histologic heterogeneity.

Each ROI corresponded to a 20× field of view (~0.69 mm^2^, 2,628,288 pixels; pixel size = 0.5112 µm × 0.5112 µm). To standardize quantification, densities were reported as cells/mm^2^, rendering them independent of the number of ROIs per case and allowing for direct inter-patient comparisons.

In parallel, total DAPI-segmented nuclei counts per ROI were extracted to calculate subset proportions, enabling relative representation analyses of immune populations. These proportions were used to complement density-based findings.

### 4.6. HLA Antibody Testing and HLA Typing

HLA antibody testing and typing methods used in this study have been described previously [32]. HLA antibody specificities were determined using the LABScreen^®^ Single Antigen Class I and Class II assays (One Lambda Inc., Canoga Park, CA, USA). Single antigen bead tests were conducted to detect antibodies against HLA-A, -B, -C, -DRB1, -DRB3, -DRB4, -DRB5, and -DQB. HLA-A, -B, -C, and DR were typed at low-to-medium resolution using the BioSewoomTM PCR/SSP kit (BioSewoom Inc., Seoul, Republic of Korea). HLA-DQB1 high-resolution typing was performed with AVITATM plus HLA-DQB1 SBT kits (BioWithus Inc., Seoul, Republic of Korea) [20].

### 4.7. Statistical Analysis

Categorical variables were reported as absolute and relative frequencies, while quantitative variables were expressed as means with standard deviations (SDs) or medians with interquartile ranges (IQRs) based on their distribution. One-way analysis of variance (ANOVA) was used to compare normally distributed variables such as age and body mass index (BMI) across different rejection types (NR, TCMR, ABMR). Non-normally distributed variables, including serum creatinine at the time of biopsy, time to biopsy, and follow-up period, were compared using the Kruskal–Wallis test. Pairwise comparisons between groups for non-normally distributed variables were performed using the Mann–Whitney U test with Bonferroni correction to account for multiple comparisons. Categorical variables were compared using the chi-square test. The area under the receiver operating characteristic curve (AUC) was calculated to evaluate the ability of specific immune cell subsets, particularly CD14^+^CD11c^+^ and CD3-PAX8-CD16^+^CD57^+^, to differentiate between ABMR, TCMR, and NR groups.

Survival analysis was performed using Cox proportional hazards regression to evaluate the associations between immune cell subsets and death-censored graft survival. The Cox model was employed to estimate hazard ratios (HRs) and their corresponding 95% confidence intervals (CIs) for the predictors, with both the CD14^+^CD11c^+^ and CD3-PAX8-CD16^+^CD57^+^ subsets included as covariates. The time to graft failure was defined as the time between the kidney transplantation and the graft loss event (death-censoring). Patients without an event at the last follow-up were censored.

The proportional hazards assumption was tested using scaled Schoenfeld residuals to confirm that the hazard ratios remained constant over time. Akaike’s Information Criterion (AIC) was calculated to compare model fit, and the significance of predictors was evaluated using Wald tests. Statistical significance was set at *p* < 0.05 for all tests. Analyses were performed using SPSS version 29.0 for Mac (IBM Corp., Chicago, IL, USA), GraphPad Prism version 10.2.3 (GraphPad Software Inc., San Diego, CA, USA), and Python 3.9 in a Jupyter Notebook environment (Anaconda distribution).

## 5. Conclusions

In conclusion, our study explored the diagnostic potential of immune cell subsets in kidney transplant biopsies, with particular focus on CD14^+^CD11c^+^ monocytes/macrophages and CD3-PAX8-CD16^+^CD57^+^ natural killer cells. The ROC curve analysis demonstrated moderate discriminatory ability between ABMR, TCMR, and NR groups. A trend towards a significant association between the CD3-PAX8-CD16^+^CD57^+^ subset and graft survival was observed after kidney transplantation. These findings suggest that CD14^+^CD11c^+^ and CD3-PAX8-CD16^+^CD57^+^ cells may play a role in graft injury, warranting further studies with larger cohorts to better understand their potential as biomarkers in kidney transplantation. These findings contribute to the growing body of evidence supporting the use of immune cell profiling as a tool for enhancing diagnostic precision and guiding therapeutic decisions in kidney transplantation.

## Figures and Tables

**Table 1:** Baseline Characteristics of Kidney Transplant Recipients by Rejection Type.

Variables	Total (n = 38)	NR (n = 7)	TCMR (n = 12)	ABMR (n = 19)	*p*-Value	Pairwise Comparisons (Bonferroni)
Mean Age, y (SD)	43.4 (13.3)	45.3 (14.2)	46.0 (13.2)	41.1 (13.3)	0.562	
Female Sex, n (%)	7 (18.4)	1 (14.3)	2 (16.7)	4 (21.1)	0.909	
Body mass index, kg/m^2^ (SD)	22.5 (2.6)	23.3 (2.5)	21.4 (2.7)	22.8 (2.4)	0.252	
Deceased donor, n (%)	11 (28.9)	1 (14.3)	3 (25)	7 (36.8)	0.497	
Previous Transplant, n (%)	8 (21.1)	1 (14.3)	0	7 (36.8)	0.044	NR vs. ABMR (*p* = 0.0784), NS for others
Time to Biopsy, month (SD)	27.3 (24.4)	9.6 (7.5)	21.4 (15.5)	37.6 (28.3)	0.01	NR vs. ABMR (*p* = 0.0107), NS for others
Cause of ESRD, n (%)						
HPT	4 (10.5)	1 (16.7)	0	3 (15.0)		
DM	6 (15.8)	1 (16.7)	3 (37.5)	2 (10.0)		
GN	6 (15.8)	2 (33.3)	1 (12.5)	3 (15.0)		
Other	4 (10.5)	0	1 (12.5)	3 (15.0)		
Unknown	18 (47.4)	3 (50.0)	7 (87.5)	8 (40.0)		
ABO-incompatible KT, n (%)	9 (23.7)	3 (42.9)	2 (16.7)	4 (21.1)	0.402	
Serum Creatinine at the time of Biopsy, mg/dL (SD)	2.8 (1.8)	2.6 (1.3)	2.5 (1.1)	3.1 (2.3)	0.98	
PRA > 10%, n (%)	15 (41.7)	4 (57.1)	3 (27.3)	8 (44.4)	0.431	
DSA at the time of Biopsy, n (%)	19 (50.0)	5 (71.4)	2 (16.7)	12 (63.2)	0.019	NR vs. ABMR (*p* = 0.0734), NS for others
CNI at the time of Biopsy, n (%)					0.269	
Cyclosporine	10 (27.8)	1 (14.3)	5 (45.5)	4 (22.2)		
Tacrolimus	26 (72.2)	6 (85.7)	6 (54.5)	14 (77.8)		
Follow-up after biopsy, month (SD)	63.7 (43.1)	63.2 (41.6)	74.3 (47.7)	57.1 (41.6)	0.339	NS

NR, no rejection; TCMR, T-cell-mediated rejection; ABMR, antibody-mediated rejection; SD, standard deviation; ESRD, end-stage renal disease; HPT, Hypertension; DM, Diabetes Mellitus; GN, Glomerular Nephropathy; KT, kidney transplantation; PRA, panel-reactive antibody; DSA, donor specific antibody; CNI, calcineurin inhibitor. NS denotes non-significant comparisons (*p* ≥ 0.05) based on chi-square or Kruskal–Wallis tests. Pairwise comparisons were performed with Fisher’s exact test (categorical variables) and the Mann–Whitney U test (continuous variables) with Bonferroni correction applied.

**Table 2:** Histopathological Characteristics of Kidney Transplant Recipients by Rejection Type.

Variables	Total (n = 38)	NR (n = 7)	TCMR (n = 12)	ABMR (n = 19)	*p*-Value	Pairwise Comparisons (Bonferroni)
C4d staining, n (%)					0.915	
<10%	34 (89.5)	6 (85.7)	12 (100.0)	16 (84.2)		
≥10%, <50%	3 (7.9)	1 (14.3)	0 (0.0)	2 (10.5)		
≥50%	0 (0.0)	0 (0.0)	0 (0.0)	0 (0.0)		
IFTA, n (%)					0.106	
Minimal	7 (18.4)	3 (42.9)	2 (16.7)	2 (10.5)		
Mild	17 (44.7)	2 (28.6)	8 (66.7)	7 (36.8)		
Moderate-to-Severe	14 (36.8)	2 (28.6)	2 (16.7)	10 (52.6)		
Mean Banff score, Mean (SD)						
i	1.58 (1.11)	1.29 (1.38)	1.75 (1.14)	1.58 (1.02)	0.689	
t	1.64 (1.18)	1.43 (1.27)	1.92 (0.90)	1.53 (1.01)	0.513	
ti	2.00 (0.94)	1.57 (1.13)	2.17 (0.84)	2.06 (0.94)	0.401	
v	0.08 (0.27)	0.00 (0.00)	0.17 (0.39)	0.05 (0.23)	0.379	
g	0.82 (0.93)	0.57 (0.79)	0.45 (0.93)	1.10 (0.97)	0.151	
ci	1.32 (1.07)	0.57 (0.79)	1.00 (0.83)	1.68 (1.11)	**0.017**	NR vs. ABMR (*p* = 0.024) NS for others
ct	1.24 (0.94)	0.57 (0.79)	1.00 (0.74)	1.63 (1.01)	**0.018**	NR vs. ABMR (*p* = 0.047) NS for others
cg	0.46 (0.94)	0.00 (0.00)	0.36 (0.92)	0.67 (1.08)	0.272	
mm	0.14 (0.38)	0.00 (0.00)	0.27 (0.47)	0.10 (0.38)	0.338	
cv	0.97 (0.97)	0.57 (0.54)	0.92 (1.08)	1.16 (1.02)	0.393	
ah	0.79 (1.00)	0.29 (0.49)	1.25 (1.14)	0.68 (0.95)	0.097	
ptc	1.63 (1.17)	0.79 (1.07)	1.92 (1.24)	2.32 (0.58)	**<0.001**	NR vs. ABMR (*p* = 0.017) TCMR vs. ABMR (*p* = 0.016)

This table presents the histopathological characteristics and Banff scores of kidney transplant recipients stratified by rejection type (NR, TCMR, ABMR). The Banff scores include interstitial inflammation (i), tubulitis (t), total inflammation score (ti, sum of i and t), vascular inflammation (v), glomerulitis (g), chronic interstitial fibrosis (ci), chronic tubular atrophy (ct), chronic transplant glomerulopathy (cg), mesangial matrix expansion (mm), chronic vascular intimal fibrosis (cv), arterial hyalinosis (ah), and peritubular capillaritis (ptc). *p*-values indicate statistical comparisons across rejection types using the appropriate statistical tests: Chi-square test for categorical variables (C4d staining and IFTA) and Kruskal–Wallis test for continuous variables (Banff scores). If the overall test was significant (*p* < 0.05), pairwise post hoc comparisons (Mann–Whitney U test for continuous variables, Fisher’s exact test for categorical variables) were performed. Both raw *p*-values and Bonferroni-adjusted *p*-values are reported for multiple pairwise comparisons (NR vs. TCMR, NR vs. ABMR, TCMR vs. ABMR). Significant differences (*p* < 0.05) are bolded, with Bonferroni-corrected *p*-values reported in parentheses. “NS” indicates non-significant comparisons. NR: No rejection; TCMR: T-cell-mediated rejection; ABMR: Antibody-mediated rejection; IFTA: Interstitial fibrosis and tubular atrophy; Banff score: Banff classification of allograft pathology; C4d: Complement component 4d; SD: Standard deviation.

**Table 3:** Characterization of Immune Cell Subsets Identified via Multiplex Immunofluorescence.

Marker	Cell Subset
CD14^+^	Monocytes/macrophages
CD14^+^CD11c^+^	Monocyte-derived dendritic cells
CD14^+^CD206^+^	M2 macrophages (alternatively activated macrophages)
CD14^+^CD11c^+^CD206^+^	M2 macrophages
CD3^–^PAX8^–^CD56^+^	Natural killer (NK) cells
CD3^–^PAX8^–^CD16^+^	Cytotoxic NK cells
CD3^–^PAX8^–^CD16^+^CD57^+^	Highly cytotoxic mature NK cells
CD3^–^CD16^+^CD14^+^	Non-classical monocytes
CD3^–^CD16^+^CD14^+^CD11c^+^	Inflammatory monocytes or DC-like Cells
CD3^–^CD16^+^CD14^+^CD206^+^	Intermediate Monocytes with M2-like profile

Characterization of Immune Cell Subsets Identified via Multiplex Immunofluorescence. This table summarizes the classification of mononuclear cell subsets based on marker expression profiles in kidney allograft biopsies.

**Table 4:** Sequential Staining Order and Fluorophore Assignments for Multiplex Immunofluorescence.

**Staining Step**	**Primary Antibody**	**Primary Antibody Dilution**	**TSA Fluorophore (Opal)**	**TSA Fluorophore Dilution**
1st	CD3	1:5	Opal 480	1:150
2nd	CD11c	1:500	Opal 520	1:150
3rd	CD14	1:100	Opal 540	1:150
4th	CD57	1:100	Opal 570	1:150
5th	CD56	1:100	Opal 620	1:150
6th	CD206	1:100	Opal 650	1:150
7th	PAX8	1:100	Opal 690	1:150
8th	CD16	1:100	Opal 780	1:25 (DIG 1:100) *

* DIG (Digoxigenin) Amplification for CD16 Detection: The CD16 staining step utilized DIG-based amplification, where a digoxigenin (DIG)-conjugated antibody was applied at 1:100, followed by TSA Opal 780 at 1:25. DIG labeling is often used in mIF to enhance weak antigen signals or avoid spectral overlap with directly conjugated antibodies. This method allows for improved detection specificity while maintaining signal integrity in a complex staining panel.

**Table 5:** Fluorescence Intensity Thresholds and Cell Segmentation Parameters in InForm 2.4.4.

(A) Fluorescence Intensity Thresholds for Marker Positivity.			
Opal Fluorophore	Marker	Positivity Threshold	Cellular Location
Opal 480	CD3	0.7	Membrane (M)
Opal 520	CD11c	2.0	Membrane (M)
Opal 540	CD14	5.0	Membrane (M)
Opal 570	CD57	3.0	Membrane (M)
Opal 620	CD56	1.7	Membrane (M)
Opal 650	CD206	8.0	Membrane (M)
Opal 690	PAX8	6.0	Membrane (M)
Opal 780	CD16	0.2	Membrane (M)
**(B) Cell Segmentation Parameters.**			
**Segmentation Type**	**Segmentation Standard**	**Threshold**	**Location**
Nuclear Segmentation	Standard (DAPI-based)	0.5	Nucleus (N)

## Data Availability

The original contributions presented in this study are included in the article/Supplementary Materials. Further inquiries can be directed to the corresponding author(s).

## Supplementary Materials

The following supporting information can be downloaded at https://www.mdpi.com/article/10.3390/ijms262311569/s1.
