# Supplementary material for: Multiplex Immunofluorescence Assay with Opal Reagents for Identifying Mononuclear Cell Subsets in Kidney Allograft Rejection Types
Source: Int J Mol Sci. 2025 Nov 28;26(23):11569. doi: 10.3390/ijms262311569 (PMC12692199; doi:10.3390/ijms262311569)
Supplement: Supplementary file 1 [file ijms-26-11569-s001.zip › Supplementary_Information_v2.pdf]

Supplementary Table 1 : Densities of Mononuclear Cell Subsets in Kidney Allograft Biopsies by Rejection Type (cells/mm²)
This table provides absolute density measurements for mononuclear immune cell subsets in kidney transplant biopsies, categorized by rejection type (NR, TCMR, ABMR). Values represent cells/mm² as quantified from scanned ROIs using multiplex immunofluorescence and automated image analysis.

Patient ID	Rejection Type	CD14+CD11c+	CD14+CD206+	CD14+CD11c+CD206+	CD3-PAX8-CD56+	CD3-PAX8-CD16+	CD3-PAX8-CD56+CD57+	CD3-PAX8-CD16+CD57+	CD3-CD16+CD14+	CD3-CD16+CD14+CD11c+	CD3-CD16+CD14+CD206+
1	NR	0.086	0	0	0.257	1.07	0	0	0	0	0
2	NR	0	0.127	0	0.063	0.253	0	0.042	0	0	0
3	NR	0.145	0	0	0.058	0.291	0	0	0	0	0
4	NR	1.075	2.293	0.072	0.502	8.454	0	0.215	0.573	0.072	0.072
5	TCMR	0	0	0	0.43	16.406	0	0.179	0.358	0	0
6	TCMR	0.395	0.079	0.039	0.118	5.253	0	0.197	0.118	0.039	0
7	TCMR	0.064	0.064	0.064	0	0.963	0	0	0	0	0
8	TCMR	0.446	0.203	0	0.162	5.229	0	0.365	0.203	0	0.081
9	TCMR	0	0	0	0	0.77	0	0	0	0	0
10	TCMR	0	0.047	0	0	3.921	0	0.047	0.093	0	0
11	TCMR	2.421	0.88	0.88	1.98	9.022	0	0	0	0	0
12	TCMR	0	0.187	0	1.68	1.12	0.047	0	0	0	0
13	TCMR	0	0.649	0	0.081	5.026	0	0.081	0.081	0	0
14	TCMR	0.394	0.107	0.072	1.075	1.648	0	0.143	0	0	0
15	TCMR	0	0.077	0	0.693	3.312	0.077	0	0	0	0
16	ABMR	0.053	0	0	0.106	3.293	0	0.053	0	0	0
17	ABMR	0.459	0.098	0.066	0.229	0.721	0	0.066	0	0	0
18	ABMR	0.075	0.113	0.038	0.113	29.492	0	0.864	0.15	0.038	0
19	ABMR	0.128	12.579	0	0	11.039	0	0.128	0.128	0	0
20	ABMR	3.321	2.696	0.385	2.407	56.607	0	9.916	1.155	0.337	0.193
21	ABMR	0.616	0.616	0.308	0	2.773	0	0.308	0	0	0
22	ABMR	0	1.155	0	0	366.599	0	1.155	20.024	0	0.385
23	ABMR	1.434	0.531	0.106	2.257	2.656	0	0.08	0	0	0
24	ABMR	3.173	2.299	0.253	1.471	33.29	0.023	3.449	1.701	0.322	0.161

25	ABMR	0.231	0.154	0.039	0.327	0.25	0	0	0	0	0
26	ABMR	0.847	0.077	0.077	1.617	15.711	0	1.463	0.154	0	0
27	ABMR	2.657	0.886	0.039	0.655	30.113	0.077	4.005	2.002	0.308	0.077
28	ABMR	0.21	0	0	0.07	42.989	0	6.021	0.63	0.07	0
29	ABMR	13.093	1.733	0.513	3.722	116.103	0.128	5.969	3.081	1.091	0.193
30	TCMR	1.694	4.043	0.154	0.616	21.026	0	2.195	1.001	0.347	0.077
31	ABMR	2.421	1.98	0.44	1.76	11.222	0	1.54	0	0	0
32	ABMR	0.03	0.091	0.03	0	15.373	0	0.725	0.091	0	0
33	ABMR	0.805	0.21	0.035	0.245	9.837	0	0	0.245	0.105	0
34	ABMR	4.477	5.295	0.529	0.385	63.25	0	6.113	9.868	1.203	1.588
35	NR	0	0.128	0	0.257	56.415	0	1.027	0.193	0	0
36	NR	1.781	1.059	0.144	0.096	3.947	0	0.626	0.144	0.096	0
37	NR	0	0.28	0	0.21	3.151	0	0.21	0.07	0	0
38	ABMR	3.399	4.462	0.637	0.478	52.106	0.053	5.152	1.009	0.106	0.053

Supplementary Table 2 : Comparison of Mononuclear Cell Subset Densities Among Different Rejection Types in Kidney Transplant Biopsies

The median (interquartile range) densities of immune cell subsets are presented for each rejection type: NR (no rejection), TCMR (T-cell-mediated rejection), and ABMR (antibody-mediated rejection).

Overall P-values were calculated using the Kruskal-Wallis test. A P-value of <0.05 was considered to indicate statistical significance. Pairwise comparisons between groups were performed using the Mann-Whitney U test, with significance values adjusted using the Bonferroni correction for multiple comparisons. * indicates a statistically significant difference at the corrected significance level (P < 0.0167)

Cell Subset	NR	TCMR	ABMR	Overall P-value	NR vs TCMR	NR vs ABMR	TCMR vs ABMR
CD14+CD11c+	0.09 (0.00, 1.07)	0.03 (0.00, 4.33)	0.81 (0.13, 3.17)	0.019	0.858	0.056	*0.011
CD14+CD206+	0.13 (0, 1.06)	0.09 (0.05, 0.54)	0.62 0.10, 2.30)	0.133	0.671	0.259	0.054
CD14+CD11c+CD206+	0 (0, 0.07)	0 (0, 0.07)	0.07 (0.03, 0.39)	0.063	0.625	0.042	0.089
CD3-PAX8-CD56+	0.21 (0.06, 0.26)	0.3 (0.02, 0.98)	0.33 (0.07, 1.62)	0.63	0.553	0.339	0.714
CD3-PAX8-CD16+	3.15 (0.29, 8.45)	4.47 (1.25, 8.08)	15.71 (3.29, 52.11)	0.03	0.447	0.053	0.023
CD3-PAX8-CD56+CD57+	0 (0 , 0)	0 (0 , 0)	0 (0 , 0)	0.433	0.267	0.198	0.746
CD3-PAX8-CD16+CD57+	0.21 (0, 0.63)	0.06 (0, 0.19)	1.16 (0.08, 5.15)	0.012	0.386	0.046	*0.008
CD3-CD16+CD14+	0.07 (0, 1.19)	0.04 (0, 1.82)	0.15 (0, 1.70)	0.141	0.858	0.184	0.072
CD3-CD16+CD14+CD11c+	0 (0, 0.07)	0 (0 , 0)	0 (0, 0.31)	0.17	0.554	0.261	0.088
CD3-CD16+CD14+CD206+	0 (0 , 0)	0 (0 , 0)	0 (0, 0.16)	0.249	0.79	0.216	0.18

Supplementary Table 3: Immune Cell Subset Proportions Relative to Total DAPI^{+} Segmented Cells

ID	Rejection Type	Total Segmented Cells	CD3+ %	CD14+ %	CD14+CD11c+ %	CD14+CD206+ %	CD14+CD11c+CD206+ %	CD3-PAX8-CD56+ %	CD3-PAX8-CD16+ %	CD3-PAX8-CD56+CD57+ %	CD3-PAX8-CD16+CD57+ %	CD3-CD16+CD14+ %	CD3-CD16+CD14+CD11c+ %	CD3-CD16+CD14+CD206+ %
1	NR	53893	2.609	0.020	0.004	0.000	0.000	0.011	0.046	0.000	0.000	0.000	0.000	0.000
2	NR	12419	0.387	0.153	0.000	0.048	0.000	0.024	0.097	0.000	0.016	0.000	0.000	0.000
3	NR	11305	2.742	0.071	0.044	0.000	0.000	0.018	0.088	0.000	0.000	0.000	0.000	0.000
4	NR	57922	7.410	1.467	0.052	0.110	0.003	0.024	0.407	0.000	0.010	0.028	0.003	0.003
5	TCMR	48487	11.224	0.130	0.000	0.000	0.000	0.025	0.945	0.000	0.010	0.021	0.000	0.000
6	TCMR	58379	11.701	0.110	0.017	0.003	0.002	0.005	0.228	0.000	0.009	0.005	0.002	0.000
7	TCMR	31576	3.728	0.057	0.003	0.003	0.003	0.000	0.048	0.000	0.000	0.000	0.000	0.000
8	TCMR	34227	4.689	0.798	0.032	0.015	0.000	0.012	0.377	0.000	0.026	0.015	0.000	0.006
9	TCMR	9745	13.094	0.010	0.000	0.000	0.000	0.000	0.041	0.000	0.000	0.000	0.000	0.000
10	TCMR	30066	5.391	0.153	0.000	0.003	0.000		0.279	0.000	0.003	0.007	0.000	0.000
11	TCMR	96814	3.234	0.011	0.011	0.004	0.004	0.009	0.042	0.000	0.000	0.000	0.000	0.000
12	TCMR	37744	1.322	0.538	0.000	0.011	0.000	0.095	0.064	0.003	0.000	0.000	0.000	0.000
13	TCMR	21229	7.895	0.334	0.000	0.038	0.000	0.005	0.292	0.000	0.005	0.005	0.000	0.000
14	TCMR	52151	9.354	0.222	0.021	0.006	0.004	0.058	0.088	0.000	0.008	0.000	0.000	0.000

15	TCMR	34583	7.816	0.072	0.000	0.003	0.000	0.026	0.124	0.003	0.000	0.000	0.000	0.000
16	ABMR	22288	7.040	0.090	0.004	0.000	0.000	0.009	0.278	0.000	0.004	0.000	0.000	0.000
17	ABMR	43108	3.197	1.011	0.032	0.007	0.005	0.016	0.051	0.000	0.005	0.000	0.000	0.000
18	ABMR	58670	5.921	0.063	0.003	0.005	0.002	0.005	1.338	0.000	0.039	0.007	0.002	0.000
19	ABMR	10093	2.705	4.340	0.010	0.971	0.000	0.000	0.852	0.000	0.010	0.010	0.000	0.000
20	ABMR	38159	6.444	1.981	0.181	0.147	0.021	0.131	3.082	0.000	0.540	0.063	0.018	0.010
21	ABMR	76524	21.319	0.260	0.008	0.008	0.004	0.000	0.035	0.000	0.004	0.000	0.000	
22	ABMR	4619	14.310	1.645	0.000	0.065	0.000	0.000	20.611	0.000	0.065	1.126	0.000	0.022
23	ABMR	100786	13.263	1.079	0.054	0.020	0.004	0.084	0.099	0.000	0.003	0.000	0.000	
24	ABMR	85326	6.274	2.367	0.162	0.117	0.013	0.075	1.697	0.001	0.176	0.087	0.016	0.008
25	ABMR	104649	4.077	0.211	0.011	0.008	0.002	0.016	0.012	0.000	0.000	0.000	0.000	
26	ABMR	20256	3.846	0.592	0.054	0.005	0.005	0.104	1.007	0.000	0.094	0.010	0.000	
27	ABMR	61158	11.148	1.859	0.113	0.038	0.002	0.028	1.279	0.003	0.170	0.085	0.013	0.003
28	ABMR	38027	7.879	0.607	0.008	0.000	0.000	0.003	1.615	0.000	0.226	0.024	0.003	
29	ABMR	49649	23.525	1.269	0.411	0.054	0.016	0.117	3.644	0.004	0.187	0.097	0.034	0.006
30	TCMR	68443	6.471	1.788	0.064	0.153	0.006	0.023	0.798	0.000	0.083	0.038	0.013	0.003
31	ABMR	9288	5.523	2.466	0.118	0.097	0.022	0.086	0.549	0.000	0.075	0.000	0.000	
32	ABMR	68859	5.569	0.030	0.001	0.004	0.001	0.000	0.739	0.000	0.035	0.004	0.000	
33	ABMR	70037	21.589	0.286	0.033	0.009	0.001	0.010	0.401	0.000	0.000	0.010	0.004	
34	ABMR	55247	14.918	2.532	0.168	0.199	0.020	0.014	2.378	0.000	0.230	0.371	0.045	0.060

35	NR	30303	2.525	0.026	0.000	0.007	0.000	0.013	2.901	0.000	0.053	0.010	0.000	
36	NR	30512	8.013	1.170	0.121	0.072	0.010	0.007	0.269	0.000	0.043	0.010	0.007	
37	NR	61630	5.294	0.339	0.000	0.006	0.000	0.005	0.073	0.000	0.005	0.002	0.000	
38	ABMR	39417	14.798	1.832	0.162	0.213	0.030	0.023	2.489	0.003	0.246	0.048	0.005	0.003

Supplementary Table 4: $CD3^+$ T Cell Counts, ROI Areas, and Densities Across Rejection Types

This table presents total $CD3^+$ T cell counts, ROI imaging parameters, and computed cell densities (cells/mm²) for each case. $CD3^+$ cell identification was based on normalized fluorescence intensity thresholds from multiplex segmentation data. ROI area was calculated as the product of ROI number and 0.69 mm² (the area per scanned region). $CD3^+$ T cell densities were computed by dividing $CD3^+$ counts by total ROI area. Rejection types include ABMR (antibody-mediated rejection), TCMR (T-cell-mediated rejection), and NR (no morphologic evidence of active rejection).

Sample ID	Rejection Type	Number of ROI	Total ROI Area (mm^2)	$CD3^+$ T Cell Count	$CD3^+$ T Cell Density (cells/mm^2)
1	NR	24	16.56	1406	84.903
2	NR	6	4.14	48	11.594
3	NR	7	4.83	310	64.182
4	NR	25	17.25	4292	248.812
5	TCMR	22	15.18	5442	358.498
6	TCMR	25	17.25	6831	396.000
7	TCMR	13	8.97	1177	131.215
8	TCMR	14	9.66	1605	166.149
9	TCMR	15	10.35	1276	123.285
10	TCMR	12	8.28	1621	195.773
11	TCMR	17	11.73	3131	266.922

12	TCMR	23	15.87	499	31.443
13	TCMR	9	6.21	1676	269.887
14	TCMR	22	15.18	4878	321.344
15	TCMR	15	10.35	2703	261.159
16	ABMR	15	10.35	1569	151.594
17	ABMR	16	11.04	1378	124.819
18	ABMR	22	15.18	3474	228.854
19	ABMR	9	6.21	273	43.961
20	ABMR	14	9.66	2459	254.555
21	ABMR	31	21.39	16314	762.693
22	ABMR	3	2.07	661	319.324
23	ABMR	29	20.01	13367	668.016
24	ABMR	24	16.56	5353	323.249
25	ABMR	30	20.7	4267	206.135
26	ABMR	8	5.52	779	141.123
27	ABMR	25	17.25	6818	395.246
28	ABMR	16	11.04	2996	271.377
29	ABMR	15	10.35	11680	1128.502
30	TCMR	26	17.94	4429	246.878
31	ABMR	5	3.45	513	148.696
32	ABMR	21	14.49	3835	264.665
33	ABMR	22	15.18	15120	996.047
34	ABMR	19	13.11	8242	628.680
35	NR	14	9.66	765	79.193
36	NR	11	7.59	2445	322.134

37	NR	21	14.49	3263	225.190
38	ABMR	15	10.35	5833	563.575

Supplementary Table 5: Kruskal–Wallis and Post Hoc Dunn's Tests Comparing Immune Subset Proportions

Subset	Kruskal_p	Comparison	Dunn_p_adj
**CD3+_%**	0.0447	**ABMR vs NR**	***0.0384***
CD3+_%	0.0447	ABMR vs TCMR	1.0000
CD3+_%	0.0447	NR vs TCMR	0.3293
CD14+_%	0.0133	ABMR vs NR	0.1297
**CD14+_%**	0.0133	**ABMR vs TCMR**	***0.0225***
CD14+_%	0.0133	NR vs TCMR	1.0000
CD14+CD11c+_%	0.0177	ABMR vs NR	0.3269
**CD14+CD11c+_%**	0.0177	**ABMR vs TCMR**	***0.0186***
CD14+CD11c+_%	0.0177	NR vs TCMR	1.0000
CD14+CD206+_%	0.1006	ABMR vs NR	1.0000
CD14+CD206+_%	0.1006	ABMR vs TCMR	0.1009
CD14+CD206+_%	0.1006	NR vs TCMR	1.0000
CD14+CD11c+CD206+_%	0.0356	ABMR vs NR	0.1101
CD14+CD11c+CD206+_%	0.0356	ABMR vs TCMR	0.1043
CD14+CD11c+CD206+_%	0.0356	NR vs TCMR	1.0000
CD3-PAX8-CD56+_%	0.9130	ABMR vs NR	1.0000
CD3-PAX8-CD56+_%	0.9130	ABMR vs TCMR	1.0000
CD3-PAX8-CD56+_%	0.9130	NR vs TCMR	1.0000
CD3-PAX8-CD16+_%	0.0269	ABMR vs NR	0.2401
**CD3-PAX8-CD16+_%**	0.0269	**ABMR vs TCMR**	***0.0376***
CD3-PAX8-CD16+_%	0.0269	NR vs TCMR	1.0000
CD3-PAX8-CD56+CD57+_%	0.4373	ABMR vs NR	0.5991

CD3-PAX8-CD56+CD57+_%	0.4373	ABMR vs TCMR	1.0000
CD3-PAX8-CD56+CD57+_%	0.4373	NR vs TCMR	1.0000
CD3-PAX8-CD16+CD57+_%	0.0229	ABMR vs NR	0.4941
**CD3-PAX8-CD16+CD57+_%**	0.0229	**ABMR vs TCMR**	***0.0213***
CD3-PAX8-CD16+CD57+_%	0.0229	NR vs TCMR	1.0000
CD3-CD16+CD14+_%	0.1590	ABMR vs NR	0.5633
CD3-CD16+CD14+_%	0.1590	ABMR vs TCMR	0.2433
CD3-CD16+CD14+_%	0.1590	NR vs TCMR	1.0000
CD3-CD16+CD14+CD11c+_%	0.1631	ABMR vs NR	0.8973
CD3-CD16+CD14+CD11c+_%	0.1631	ABMR vs TCMR	0.1946
CD3-CD16+CD14+CD11c+_%	0.1631	NR vs TCMR	1.0000
CD3-CD16+CD14+CD206+_%	0.0361	ABMR vs NR	0.9759
CD3-CD16+CD14+CD206+_%	0.0361	ABMR vs TCMR	0.3153
CD3-CD16+CD14+CD206+_%	0.0361	NR vs TCMR	1.0000

Supplementary Table 6. Immune Cell Subset Proportions by Rejection Category: Summary Statistics (% of Total Cells)

Immune Subset	Rejection_Type	n	Mean (%)	SD (%)	Q1 (25th %)	Q3 (75th %)
CD14+CD11c+CD206+_%	ABMR	19	0	0	0	0
CD14+CD11c+CD206+_%	NR	7	0	0	0	0
CD14+CD11c+CD206+_%	TCMR	12	0	0	0	0
CD14+CD11c+_%	ABMR	19	0.1	0.1	0	0.1
CD14+CD11c+_%	NR	7	0	0	0	0
CD14+CD11c+_%	TCMR	12	0	0	0	0
CD14+CD206+_%	ABMR	19	0.1	0.2	0	0.1
CD14+CD206+_%	NR	7	0	0	0	0.1
CD14+CD206+_%	TCMR	12	0	0	0	0
CD14+_%	ABMR	19	1.3	1.1	0.3	1.9
CD14+_%	NR	7	0.5	0.6	0	0.8
CD14+_%	TCMR	12	0.4	0.5	0.1	0.4
CD3+_%	ABMR	19	10.2	6.5	5.5	14.6

CD3+_%	NR	7	4.1	2.6	2.6	6.4
CD3+_%	TCMR	12	7.2	3.5	4.4	9.8
CD3-CD16+CD14+CD11c+_%	ABMR	19	0	0	0	0
CD3-CD16+CD14+CD11c+_%	NR	7	0	0	0	0
CD3-CD16+CD14+CD11c+_%	TCMR	12	0	0	0	0
CD3-CD16+CD14+CD206+_%	ABMR	11	0	0		
CD3-CD16+CD14+CD206+_%	NR	4	0	0		
CD3-CD16+CD14+CD206+_%	TCMR	12	0	0	0	0
CD3-CD16+CD14+_%	ABMR	19	0.1	0.3	0	0.1
CD3-CD16+CD14+_%	NR	7	0	0	0	0
CD3-CD16+CD14+_%	TCMR	12	0	0	0	0
CD3-PAX8-CD16+CD57+_%	ABMR	19	0.1	0.1	0	0.2
CD3-PAX8-CD16+CD57+_%	NR	7	0	0	0	0
CD3-PAX8-CD16+CD57+_%	TCMR	12	0	0	0	0
CD3-PAX8-CD16+_%	ABMR	19	2.2	4.5	0.3	2
CD3-PAX8-CD16+_%	NR	7	0.6	1	0.1	0.3
CD3-PAX8-CD16+_%	TCMR	12	0.3	0.3	0.1	0.3
CD3-PAX8-CD56+CD57+_%	ABMR	19	0	0	0	0
CD3-PAX8-CD56+CD57+_%	NR	7	0	0	0	0
CD3-PAX8-CD56+CD57+_%	TCMR	12	0	0	0	0
CD3-PAX8-CD56+_%	ABMR	19	0	0	0	0.1
CD3-PAX8-CD56+_%	NR	7	0	0	0	0
CD3-PAX8-CD56+_%	TCMR	11	0	0		

Supplementary Table 7: Banff Scores for Individual Cases in the Study

This table presents the Banff histopathological scores for each case, categorized by rejection type: NR (No Rejection), TCMR (T-cell-mediated rejection), and ABMR (Antibody-mediated rejection). Scoring parameters include: i (interstitial inflammation), t (tubulitis), ti (total inflammation), v (intimal arteritis), g (glomerulitis), ci (interstitial fibrosis), ct (tubular atrophy), cg (transplant glomerulopathy), mm (mesangial matrix expansion), cv (vascular intimal fibrosis), ah (arterial hyalinosis), ptc (peritubular capillaritis), C4d Staining (immunohistochemical marker for antibody-mediated rejection), and aah (acute antibody-mediated arteritis).

Patient ID	Rejection Type	i	t	ti	v	g	ci	ct	cg	mm	cv	ah	ptc	C4d	aah
1	NR	3	0	3	0	2	0	0	0	0	0	0	2	0	0
2	NR	0	0	0	0	0	0	0	0	0	0	0	0	0	0
3	NR	1	1	1	1	0	0	0	0	0	0	0	0	0	0
4	NR	0	1	0	0	1	0	1	0	0	0	0	0	1	0
5	TCMR	3	3	3	1	0	0	0	0	0	0	2	2	0	2
6	TCMR	3	2	2	0	0	0	1	0	0	1	1	2	0	0
7	TCMR	1	1		0	0	1	1	0	0	3	3	0	0	3
8	TCMR	0	1	1	0	0	0	0	0	1	1	1	0	1	0
9	TCMR	0	1	1	0	0	1	1	0	0	0	0	0	0	0
10	TCMR	3	3	3	0		1	1			0	0	3	1	0
11	TCMR	2	3	2	0	0	1	1	0	1	0	2	0	0	2
12	TCMR	2	3	3	1	3	0	1	0	0	1	0	1	1	0
13	TCMR	1	1	1	0	0	1	1	0	0	1	0	0	0	0
14	TCMR	2	2	2	0	0	1	1	0	0	1	2	0	0	2
15	TCMR	1	1	3	0	1	3	3	1	1	3	3	0	0	2
16	ABMR	0	0	1	2	2	1	1	0	0	1	0	2	0	0
17	ABMR	0	0	0	0	1	0	1	0	0	2	0	2	0	0

18	ABMR	3	3	3	0	1	1	1	0	0	0	0	3	1	0
19	ABMR	1	1	2	0	0	3	1	2	1	1	2	2	3	2
20	ABMR	1	1	2	0	1	1	1	0	0	1	2	3	0	0
21	ABMR	3	3	3	0	1	2	2	0	0	0	0	2	0	0
22	ABMR	3	2	3	0	0	0	1	0	0	0	0	3	0	0
23	ABMR	2	1	3	0	0	3	3	0	0	2	1	1	1	1
24	ABMR	2	1	2	0	2	1	1	1	1	1	1	3	0	0
25	ABMR	1	1	2	0	2	2	2	3	0	2	3	3	0	3
26	ABMR	1	2	2	0	1	1	1	0	0	2	0	2	1	0
27	ABMR			1	0	0	3	3	3		3	0	2	2	
28	ABMR	2	1	2	1	2	0	0	0	0	1	1	2	0	0
29	ABMR	3	3	3	0	1	2	2	0	0	0	0	2	0	0
30	TCMR	3	2	3	0	1	1	1	3	0	0	1	3	0	0
31	ABMR	1	1	1	0	2	1	0	0	0	0	0	3	0	0
32	ABMR	1		1	0	1	3	3	0	0	1	1	2	0	0
33	ABMR	1	1	2	0	x	3	3	x	0	0	0	3	0	0
34	ABMR	3	3	3	0	2	2	2	1	1	3	2	2	2	2
35	NR	3	3	3	0	0	2	1	0	0	1	1	2	2	1
36	NR	2	3	2	0	1	1	1	0	0	1	1	3	1	0
37	NR	0	2	1	0	0	1	1	0	0	1	0	0	0	0
38	ABMR	1	2	3	0	0	3	3	1	0	0	0	2	0	0

Supplementary Table 8 : Reagents and Buffers Used in Multiplex Immunofluorescence and Antigen Retrieval

Purpose	Name	Vendor	Catalog No.
Dewax	Leica Bond Dewax Solution	Leica Biosystems	#AR9222
Antigen retrieval	Bond Epitope Retrieval 2	Leica Biosystems	#AR9640
Blocking	Antibody Diluent/Block	AKOYA Biosciences	ARD1001EA
Secondary antibody	Opal Polymer HRP Ms+Rb	AKOYA Biosciences	ARH1001EA
Antigen retrieval	Bond Epitope Retrieval 1	Leica Biosystems	#AR9961

Supplementary Table 9: Reagent Volumes and Incubation Times for Multiplex Immunofluorescence

Step	Solution	Volume per Slide (µL)	Incubation Time	Temperature
1 (Dewaxing & Hydration)	Bond Dewax Solution	150 µL	30s	72°C
	Alcohol	150 µL	Immediate	RT
2 (Antigen Retrieval)	ER2 (pH 9.0)	150 µL	30m	100°C
3 (Blocking & Staining)	Antibody diluent/block	150 µL	5m	RT
	Primary antibody	150 µL	30m	RT
	Opal Polymer HRP Ms+Rb	150 µL	10m	RT
	TSA Fluorophore	150 µL	10m	RT
4 (Heat-Induced Epitope Retrieval for Multiplexing)	ER1 (pH 6.0)	150 µL	20m	95°C
5 (Counterstaining & Mounting)	DAPI	150 µL	5m	RT

Supplementary Table 10: Antibody Panel Used for Multiplex Immunofluorescence Staining

Marker	Clone	Source	Catalog Number	Dilution
CD3	Rabbit monoclonal	Ventana	790-4341	1:5
CD11c	Rabbit monoclonal	Abcam	Ab52632	1:500
CD14	Rabbit monoclonal	Abcam	Ab183322	1:100
CD57	Mouse monoclonal (NK1)	Invitrogen	MA5-12008	1:100
CD56	Mouse monoclonal (clone CD564)	Leica Biosystem	CD56-504-L-CE	1:100
CD206	Rat monoclonal	R&D systems,	MAB2534	1:100
PAX8	MRQ-50	Cell Marque	363M-16	1:100
CD16	Rabbit monoclonal	Cell marque	116R	1:100
